# Squat and countermovement jump performance across a range of loads: a comparison between Smith machine and free weight execution modes in elite sprinters

**DOI:** 10.5114/biolsport.2022.112085

**Published:** 2022-01-25

**Authors:** Irineu Loturco, Michael R. Mcguigan, Tomás T. Freitas, Fábio Y. Nakamura, Daniel A. Boullosa, Pedro L. Valenzuela, Lucas A. Pereira, Fernando Pareja-Blanco

**Affiliations:** 1 NAR – Nucleus of High Performance in Sport, São Paulo, Brazil; 2 Department of Human Movement Sciences, Federal University of São Paulo, São Paulo, Brazil; 3 University of South Wales, Pontypridd, Wales, United Kingdom; 4 Sports Performance Research Institute New Zealand (SPRINZ), Auckland University of Technology, Auckland, New Zealand; 5 School of Medical and Health Sciences, Edith Cowan University, Perth, Australia; 6 UCAM Research Center for High Performance Sport, Catholic University of Murcia, Murcia, Spain; 7 Faculty of Sport Sciences, Catholic University of Murcia (UCAM), Murcia, Spain; 8 Research Center in Sports Sciences, Health Sciences and Human Development (CIDESD), University Institute of Maia (ISMAI), Maia, Portugal; 9 Associate Graduate Program in Physical Education Universidade de Pernambuco (UPE)/Universidade Federal da Paraíba (UFPB), João Pessoa, Brazil; 10 College of Healthcare Sciences, Campus Townsville, James Cook University, Townsville, Australia; 11 INISA, Graduate Program of Movement Sciences, Campus Campo Grande, Federal University of Mato Grosso do Sul, Campo Grande, Mato Grosso do Sul, Brazil; 12 Research and Development Department, iLOAD Solutions, Campo Grande, Mato Grosso do Sul, Brazil; 13 Faculty of Sport Sciences, Universidad Europea de Madrid, Madrid, Spain; 14 Department of Sport and Health, Spanish Agency for Health Protection in Sport (AEPSAD), Madrid, Spain; 15 Physical Performance & Sports Research Center, Pablo de Olavide University, Seville, Spain

**Keywords:** Athletic performance, Track and field, Vertical jump, Loaded jump, Resistance training, Ballistic exercises

## Abstract

The aims of this study were to: 1) provide and compare the height achieved during Smith machine (SM) and free weight (FW) loaded jumps executed over a wide spectrum of loads (40–120% of body mass [BM]); and 2) test the difference between loaded and unloaded squat jump (SJ) and countermovement jump (CMJ) attempts in ten highly trained male sprinters. On the first visit, athletes performed unloaded SJ and CMJ, loaded SJ with loads corresponding to 40, 60, 80, 100, and 120% BM, and loaded CMJ at 100% BM using an Olympic barbell (FW). On the second visit, they performed loaded SJ and CMJ tests under the same loading conditions on the SM device and, subsequently, a half-squat one-repetition maximum (1RM) assessment. The relative strength (RS = 1RM/BM) of the athletes was 2.54 ± 0.15. Loaded SJ performance was similar between SM and FW, and across all loading conditions. Differences in favour of CMJ (higher jump heights compared with SJ) were superior in the unloaded condition but decreased progressively as a function of loading. In summary, sprinters achieved similar SJ heights across a comprehensive range of loads, regardless of the execution mode (FW or SM). The positive effect of the countermovement on jump performance is progressively reduced with increasing load.

## INTRODUCTION

Ballistic exercises are widely used by strength and conditioning coaches and practitioners of different sports [1–4]. The massive use of these exercises is directly related to their proven effectiveness and greater levels of transference to athletic performance [2, 5, 6]. Overall, ballistic lifts allow athletes to accelerate throughout the entire range of motion to the point of projection or take-off, while avoiding deceleration during the concentric phase [2, 5]. Consequently, these movements are very similar to many sport-specific tasks (e.g., throwing, punching, or jumping), where athletes have to reach higher velocities in the final portions of motor actions in order to achieve superior performance outcomes [1, 2].

Among numerous ballistic exercises, the loaded jump is one of the preferred and most frequently used [7]. The simple characteristics of this “low-cost” exercise, associated with its easy applicability and high efficiency, contribute to make loaded jumps a popular option for coaches from different sports and countries [4, 7–9]. Indeed, several studies have confirmed the effectiveness of this explosive exercise in improving numerous performance parameters, especially sprint and jump capacities [1, 8, 10, 11]. For example, Loturco et al. [10] compared two different velocity-oriented training schemes by either increasing or decreasing (i.e., a 20% increase or decrease in bar velocity as compared with the bar velocity achieved under unloaded conditions, respectively) the loaded squat jump (SJ) velocity over a 6-week training period in elite young soccer players. In general, both strategies were able to improve speed and power performance; nonetheless, the “increased velocity group” showed greater improvements in sprint speed at 5, 10, and 20 m. Similarly, McBride et al. [11] demonstrated that 8 weeks of SJ training using either light (30% of one repetition-maximum [1RM]) or heavy loads (80% 1RM) applied to “athletic subjects” (i.e., males with 2 to 4 years of resistance training experience) resulted in similar increases in 1RM squat strength, although a trend toward enhanced sprint adaptations was observed in the “light-load group”.

Additional studies have compared the performance obtained by different athletes during the SJ exercise executed at distinct loading conditions, with a special focus on elite sprinters. McBride et al. [12] reported that sprinters with a relative strength (RS; 1RM/body mass [BM]) of ~2.65 achieved, on average, jump heights equal to 23.9 and 14.1 cm at 30 and 60%1RM (or at ~80, and 160% BM), respectively. In a reference study on loaded SJ, Loturco et al. [13] reported the same values for elite sprinters, who achieved, on average, 23.4 cm during SJ trials executed at 80% BM. A previous study also examined the height achieved by top-level sprinters and jumpers during loaded countermovement jumps (CMJs) and revealed that at 40–45% 1RM, these athletes were able to jump between 20.9 and 22.5 cm [14]. These data also agree with those published by Loturco et al. [13] (20.3 cm for SJ at 100% BM or 42% 1RM; RS = 2.38), despite methodological differences in jump testing procedures (i.e., loaded SJ versus loaded CMJ). However, it is worth noting that in heavy-load (low-velocity) conditions (i.e., 100% BM), the potentiation effect of the stretch-shortening cycle may be greatly compromised, which possibly reduces its effects on jumping performance [15, 16].

Curiously, all studies providing reference values for loaded jumps for sprinters were conducted on a Smith machine (SM) device and none of them assessed these athletes using free weight (FW) SJ [7, 13]. In addition, no studies simultaneously reported and compared loaded jump performance at similar relative loads (e.g., 0 and 100% BM) when executing either SJ or CMJ. Therefore, the aims of this study were to: 1) provide and compare the height achieved during SM and FW SJ trials executed at a wide spectrum of loads (40, 60, 80, 100, and 120% BM), and 2) test the difference between unloaded and loaded SJ and CMJ attempts in a sample of top-level sprinters. We hypothesized that: 1) SJ heights at different loads would be similar between both execution modes (SM or FW), and 2) differences in favour of CMJ (i.e., higher jump heights compared to SJ) would be maximal at 0% BM and progressively decrease with increasing loads.

## MATERIALS AND METHODS

### Participants

Ten highly trained sprinters (27.1 ± 4.6 years; 84.5 ± 13.5 kg; 181.3 ± 7.4 cm) who regularly competed in regional, national, or international track and field events (personal best range in 100-m dash: 10.28–11.16 s) volunteered to participate in this study. Before participating in the study, athletes signed an informed consent form. The study was approved by the local ethics committee.

### Study Design

This comparative study assessed the differences between the height achieved in both SM and FW as well as in unloaded and loaded SJ and CMJ attempts. Tests were performed on two different days interspersed by at least 48 and a maximum of 72 h. On the first visit, athletes performed unloaded SJ and CMJ, loaded SJ with loads corresponding to 40, 60, 80, 100, and 120% BM, and loaded CMJ at 100% BM using an Olympic barbell (FW). On the second visit, they performed the loaded SJ and CMJ tests at the same loading conditions on the SM and, subsequently, a half-squat 1RM test. All athletes were familiarized with testing procedures due to their constant and regular practices at our facilities. Before the assessments, athletes performed a standardized warm-up including running at a moderate pace for 10 min followed by lower limb dynamic stretching exercises for 5 min and submaximal attempts of each test.

### Procedures

#### Loaded and unloaded vertical jumps

**FIG. 1:**
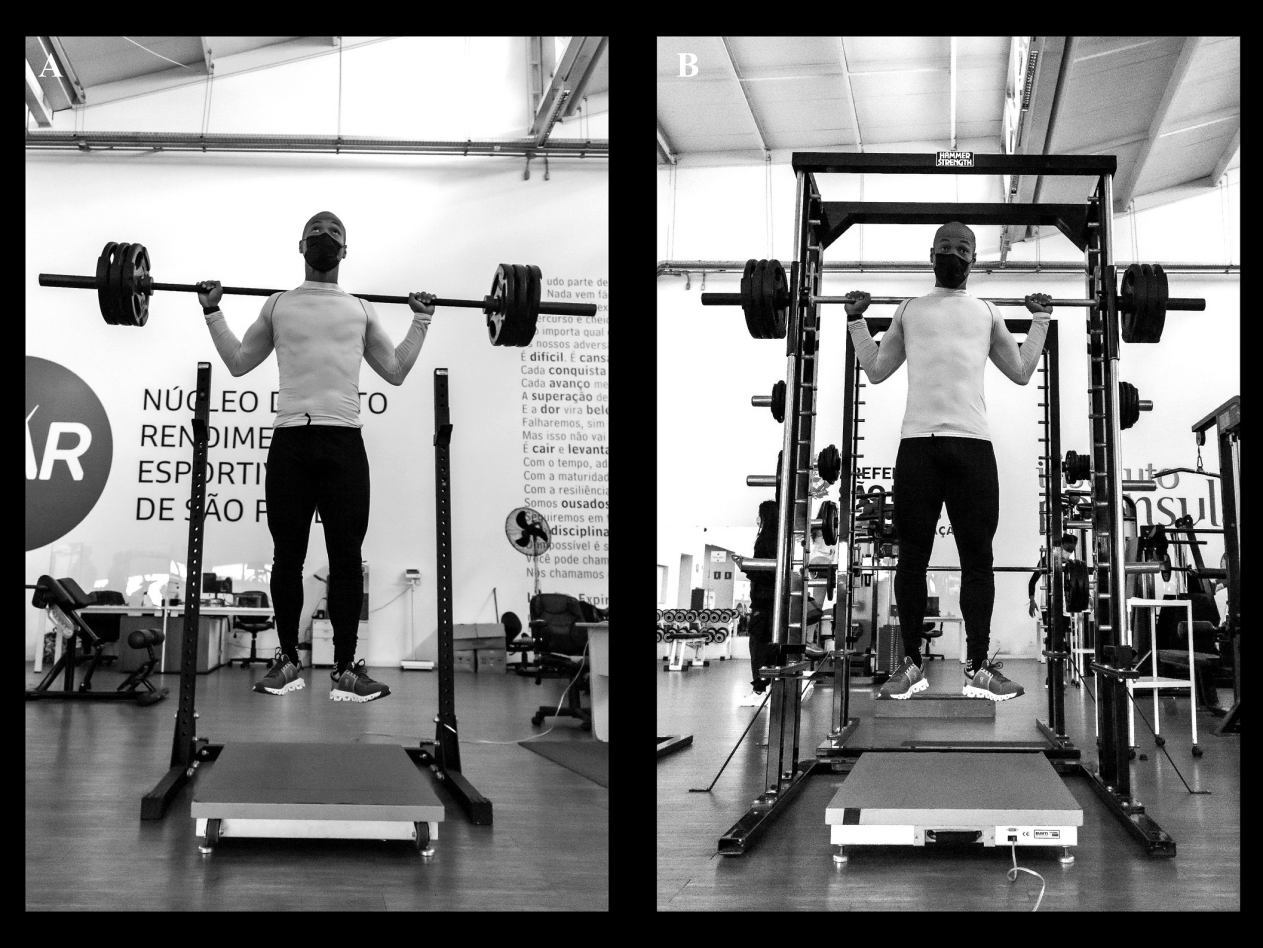
A sprinter performing free weight (A) and Smith machine (B) loaded squat jumps at 100% of body mass.

Vertical jumps were assessed using the unloaded and loaded SJ and CMJ. In the SJ, a static position with a ~90° knee flexion angle was maintained for 2 s before a jump attempt without any preparatory movement. In the CMJ, athletes were instructed to perform a downward movement followed by complete extension of the lower limbs and the amplitude of the countermovement was freely determined to avoid changes in jumping coordination. The unloaded jumps were executed with the hands on the hips. The loaded jumps were performed using an Olympic barbell or an SM device (Hammer Strength Equipment, Rosemont, IL, USA). Three attempts of each jump type were performed, interspersed by 15-s intervals. Between each trial a 3-min interval was allowed. Jump tests were performed on a force platform (AccuPower, AMTI, Watertown, MA, USA), sampling at a rate of 1,000 Hz (Figure 1, A and B). Jump height was determined by the flight time (FT) and take-off velocity (TOV) methods and the highest values obtained from each method were used for analyses.

#### Half-squat one-repetition maximum test

Maximum strength was assessed using the half-squat 1RM test, as described previously [17, 18]. Prior to the test, athletes executed a warm-up set, which consisted of 5 repetitions between 40 and 60% of the estimated 1RM. Three minutes after the warm-up, athletes were allowed up to 5 attempts at ~70, 80, 90, and > 95% of the estimated 1RM to obtain the actual 1RM value [17, 18]. A 3-min rest interval was allowed between all repetitions [17, 18]. Athletes were instructed to move the barbell as fast as possible during the concentric phase of movement in all attempts. The 1RM values were normalized by dividing the 1RM by the athletes’ BM (i.e., RS).

### Statistical Analyses

Data are presented as means ± standard deviations. Data normality was confirmed using the Shapiro-Wilk test. Differences in jump height between jump type (SJ versus CMJ), jump mode (FW versus SM), height determination method (FT versus TOV), and relative loads (from 40 to 120% BM) were determined using an analysis of variance (ANOVA) with repeated measures. When significant interactions were noted, pairwise comparisons were performed using Bonferroni’s post-hoc adjustments. The level of significance was set at *P* < 0.05. The magnitude of the differences was analysed using Cohen’s *d* effect size (ES) [19]. The ES values were interpreted using the thresholds proposed by Rhea [20] for highly trained individuals, as follows: < 0.25, 0.25–0.50, 0.50–1.00, and > 1.00 for trivial, small, moderate, and large, respectively. All tests used in this study displayed high levels of absolute and relative reliability (i.e., intraclass correlation coefficients > 0.90 and coefficients of variation < 10%).

## RESULTS

The RS of the subjects in the present study was 2.54 ± 0.15. Table 1 presents the vertical jump height for the different jump types, execution modes, and methods for jump height determination across the range of loads tested. Figure 2 depicts the comparisons between SJ heights from 40 to 120% BM, for both execution modes (FW and SM), and for both FT and TOV methods. The SJ height was progressively reduced as a function of loading, without significant differences between FW SJ and SM SJ or between the methods for vertical jump height determination (FT vs. TOV).

**TABLE 1:** Comparisons of the squat jump and countermovement jump heights between both execution modes and methods for vertical jump height determination.

	U SJ	U CMJ	ES	FW SJ 100%	FW CMJ 100%	ES	SM SJ 100%	SM CMJ 100%	ES
**FT height (cm)**	56.1 ± 5.3*^#^	60.8 ± 5.1^#^	0.72	20.4 ± 2.1	20.9 ± 3.1	0.15	21.6 ± 2.0*	22.5 ± 2.3	0.35
**TOV height (cm)**	53.7 ± 5.3*	57.8 ± 5.7	0.62	20.9 ± 2.1	21.0 ± 2.7	0.04	21.9 ± 1.8*	22.7 ± 2.3	0.31
**ES**	0.37	0.43	-	0.19	0.05	-	0.14	0.07	-

FT: flight time; TOV: take-off velocity; ES: effect-size; U: unloaded; SJ: squat jump; CMJ: countermovement jump; FW: free weight; SM: Smith-machine; 100%: load corresponding to 100% of the athletes’ body mass;

* Significant difference in relation to CMJ at the same load, *P* < 0.05;

# Significant difference comparing FT and TOV methods, *P* < 0.05.

**FIG. 2:**
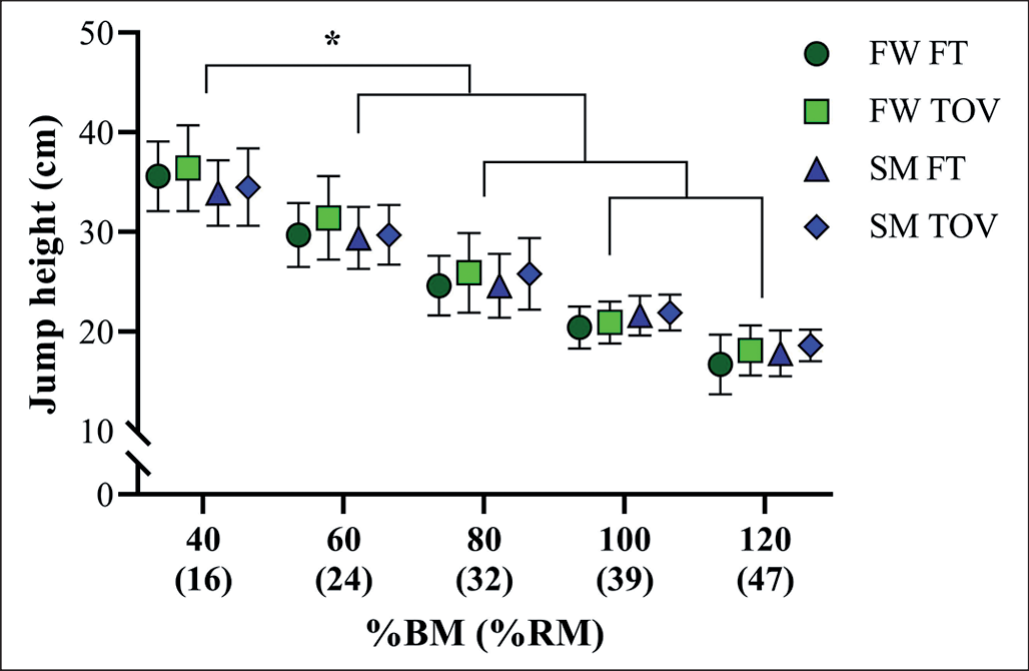
Comparisons between loaded squat jump heights from 40 to 120% of body mass, for both free weight (FW) and Smith machine (SM) device, and for both flight time (FT) and take-off velocity (TOV) methods. *Significant differences among all loading conditions, *P* < 0.05.

## DISCUSSION

The primary purpose of the current study was to compare the height achieved by top-level sprinters during SM SJ and FW SJ trials executed at a wide spectrum of loads (40–120% BM). In addition, we tested the differences between unloaded and loaded (100% BM) SJ and CMJ attempts. As expected, the SJ performance was similar between both execution modes (i.e., SM and FW), across all loading conditions. Similarly, according to our expectations, differences in favour of CMJ were maximal in the unloaded condition and decreased with increasing load (100% BM).

Sprinters achieved similar SJ heights in both SM and FW modes, from 40 to 120% BM (Figure 2; average difference of 1.08 cm, for both TOV and FT measures). These results are in line with those published by Pérez-Castilla et al. [21], who reported an average difference of 1.15 cm between SM SJ and FW SJ, using both TOV and FT procedures, across a load range of 17–75 kg. Although we can presume that the SM device could facilitate the vertical displacement of the barbell (and thus increase the vertical jump height), it appears that the FW mode allows more natural and coordinated movement of the lower extremities, which, in turn, may attenuate this “potential” jump advantage [22, 23]. This is even more prominent in toplevel sprinters, who regularly perform a substantial volume of different types of FW squat-based exercises during their resistance training routines, with different purposes and objectives (e.g., strength or power development) [24, 25]. Accordingly, our data agree with those described in a recent study conducted with an SM device [13] and confirm that, at least for a population of elite sprinters, FW SJ and SM SJ reference values may be utilized in an interchangeable manner. Strength and conditioning coaches are encouraged to use these SJ measures to compare the power-related performance of sprinters of distinct competitive levels, as well as to prescribe and control their relative training intensity through the use of the relative metrics (i.e., %BM and %1RM; Figure 2) provided here.

For this cohort of sprinters (with a mean RS equal to 2.5), 40, 60, 80, 100, and 120% BM represent, respectively, 16, 24, 32, 40, and 48% 1RM. With these relative loads, these athletes can jump, on average, from 36 to 17 cm (Figure 2). As previously mentioned, McBride et al. [12] found a similar value for loaded SJ trials executed at 80% BM (30% 1RM) in a sample of sprinters with an RS of ~2.65 (23.9 cm versus 25 cm in our sample for the same load intensity). The other measures described here are also consistent with those from two previous studies which reported the SJ performance of elite sprinters over a comprehensive range of loads (30.9 to 18.9 cm, from 40% to 110% BM) [13] or only at ~100% BM (21.3 cm) [7]. The novel finding in the present study is that, regardless of the execution mode (SM or FW), the differences in favour of CMJ (higher jump heights compared to SJ) were maximal in the unloaded condition and decreased as a function of loading (~8% difference between unloaded SJ and CMJ; ~2.4% difference between SJ and CMJ at 100% BM).

The reduced difference between SJ and CMJ at heavier loads may be due to the negative influence of excessive loading on the functionality and effectiveness of the stretch-shortening cycle [15, 16]. Indeed, heavy and very heavy loads are necessarily moved at slower velocities, requiring greater force application during longer time periods (which is even more evident in movements with relatively large ranges of motion, such as the CMJ) [15, 16]. This increased time, especially across the concentric phase, may diminish the role played by the stored elastic energy (as a result of the pre-stretching) over the intermediate and later phases of the movement [16, 26]. Accordingly, Wilson et al. [27] reported that the half-life of the stretch-shortening cycle is 0.85 seconds, and that by 1 second its potential benefits for explosive performance are reduced by 55% [26, 27]. In this context, Komi and Gollhofer [28] highlighted that “an effective stretch-shortening cycle” relies on some fundamental conditions, such as a short and fast eccentric phase followed by a rapid and immediate transition between stretch-and-shortening phases. Bosco et al. [15] reinforced this argument by suggesting that the small differences found between loaded SJ and loaded CMJ are related to the fact that, when performed with heavy loads, the CMJ “is characterized by a long stretching phase” (~500 ms). This biomechanical alteration in the movement pattern increases the transient time between eccentric and concentric phases, which greatly compromises the reutilization of the elastic energy [15].

In summary, our study demonstrated, for the first time, that sprinters achieved similar SJ heights across a comprehensive range of loads in both SM and FW execution modes. Furthermore, the difference between SJ and CMJ heights decreased with increasing loads. Thus, for example, sprinters with an RS ≥ 2.2 can jump approximately 20 cm in either SJ or CMJ trials at 100% BM (or at 40–45% 1RM), which is also in accordance with a previous study on loaded CMJ performance in high-level track and field athletes [14]. We recognize that these findings are limited by the small sample size and the very specific characteristics of the subjects (i.e., highly trained sprinters). However, our results are supported by earlier research on this topic and consolidate and generalize the reference data obtained for the SM SJ exercise [13]. Further studies are needed to test the differences between light-loaded SJ and CMJ attempts, as well to analyse the effects of loading on other jump types (e.g., drop jump) and test across different types of athletes.

## CONCLUSIONS

In a sample of elite sprinters, reference values for SM SJ and FW SJ may be utilized in an interchangeable manner. This also suggests that these top-level athletes experience similar loading magnitudes (in both absolute and relative terms) at certain percentages of BM, regardless of the execution mode. The same holds true for loaded SJ and CMJ trials executed under heavier loading conditions (e.g., 100% BM). Strength and conditioning coaches may use the relative loads presented here to monitor the resistance training sessions of their sprinters as well as to define and compare their strength-power level more precisely. Sprint coaches should be aware that, at heavier loads, the potential benefits of the stretch-shortening cycle will be lost, thus reducing the differences between SJ and CMJ performances. This will likely affect not only the movement pattern and coordination, but also the acute and chronic training responses to jump training.

## Conflict of interest declaration

The authors declared no conflict of interest.
